# Predictors of Prenatal Breastfeeding Self-Efficacy in Expectant Mothers with Gestational Diabetes Mellitus

**DOI:** 10.3390/ijerph19074115

**Published:** 2022-03-30

**Authors:** Nada Alyousefi, Arwa Alemam, Dena Altwaijri, Sarah Alarifi, Haifa Alessa

**Affiliations:** 1 Department of Family and Community Medicine, College of Medicine, King Saud University, Riyadh 11451, Saudi Arabia; 2 King Saud University Medical City, King Saud University, Riyadh 11451, Saudi Arabia; 3 College of Medicine, King Saud University, Riyadh 11451, Saudi Arabia; arwa.alemam1999@gmail.com (A.A.); altwaijridena@gmail.com (D.A.); sara1alarifi@gmail.com (S.A.); haifaalessa414@gmail.com (H.A.)

**Keywords:** breastfeeding, self-efficacy, diabetes, gestational, health education, maternal–child health services

## Abstract

Breastfeeding is beneficial for mothers with gestational diabetes mellitus (GDM). Saudi Arabia is considered one of the countries with the highest prevalence of GDM. Mothers with GDM have a low intention to breastfeed and are less likely to continue breastfeeding. This study aimed to measure breastfeeding self-efficacy among expectant mothers with GDM and quantify its determinants. This cross-sectional study recruited expectant mothers with GDM from an antenatal care clinic and queried them on breastfeeding knowledge and attitudes using the Arabic validated prenatal breastfeeding self-efficacy scale (PBSES). The study took place at the Medical City of King Saud University, during January–April 2021. The average PBSES score among 145 GDM Saudi participants was 64.07 ± 16.3. Higher academic level, previous satisfactory breastfeeding experiences, breastfeeding intention, six months or more breastfeeding experience, and health education were significantly positively correlated with PBSES score. A higher knowledge score was also correlated with a higher PBSES score (*p* = 0.002). Longer breastfeeding duration (β.197, *p* = 0.036), satisfactory previous breastfeeding experience (β.218, *p* = 0.020), and higher knowledge score (β.259, *p* = 0.004) were significant predictors of a high PBSES score. Breastfeeding self-efficacy is low among expectant Saudi mothers with GDM, especially those with unsatisfactory previous experience or low knowledge scores. Establishing systematic education about breastfeeding during antenatal care is recommended to improve breastfeeding experience and improve GDM outcomes.

## 1. Introduction

Breastfeeding (BF) is an important component of neonates’ health and provides essential nutrients. Research has demonstrated that various factors can lead to barriers to successful breastfeeding. One of these factors is gestational diabetes mellitus (GDM) [1], which is defined as “the type of glucose intolerance that develops in the second and third trimester of pregnancy, resulting in hyperglycemia of variable severity” [2].

The prevalence of GDM in Saudi Arabia is 22.9%, which is considered the third highest in Asia, with an overall incidence of 11.5%, according to a systematic review study conducted in 2017 [3]. Another large cohort study conducted in Saudi Arabia found that the prevalence of GDM in the country is 24.3%, making it one of the countries with the highest GDM prevalence [4].

There are long-term maternal consequences for women with a history of GDM, although most achieve normal blood glucose levels after delivery [5,6,7,8]. The long-term consequences include the increased risk for recurrent GDM in subsequent pregnancies, prediabetes, and overt diabetes [5]. A previous study has shown that breastfeeding reduces postpartum fasting glucose levels [6]. In addition, high-intensity breastfeeding plays a significant role in the postpartum decrease in blood glucose levels and glucose tolerance [6,7]. A retrospective cohort study showed that exclusive lactation for ≥1 month reduced the recurrence rate of GDM and possibly the risk of cardiovascular diseases in the subsequent pregnancy [9]. Evidence supports the beneficial effect of breastfeeding in postpartum, controlling blood glucose to prevent or delay the development of type 2 diabetes in women who had GDM [5,8].

Infants born to mothers with GDM are at a higher risk of being overweight; breastfeeding decreases this risk if it continues for at least three months [10]. Another critical role of breastfeeding is maintaining blood glucose levels in the normal range after delivery in infants born to women with GDM [11].

Despite all these facts, previous studies have shown that mothers with GDM have less intention to breastfeed than mothers without GDM and are less likely to continue breastfeeding [12,13]. Several barriers to adherence to breastfeeding have been identified, including lack of family support, lack of knowledge about breastfeeding benefits, maternal psychological factors, and smoking [11,14,15,16,17,18]. Cesarean section (elective or emergency), which is more prevalent among GDM mothers, has also been shown to play a significant role in delaying breastfeeding initiation, which then affects breastfeeding duration and continuation [19,20,21]. Furthermore, maternal overweight/obesity in women with GDM is strongly associated with delayed breastfeeding initiation [22]. Another study found that 40% of obese/overweight mothers never breastfed their infants compared with 20.5% of non-obese mothers [10].

Breastfeeding self-efficacy is essential for predicting breastfeeding initiation, duration, and continuation [23]. A study conducted in Saudi Arabia concluded that the average breastfeeding self-efficacy score was 70 ± 11.9 (range: 20–100) in healthy pregnant women without GDM and that there was a significant correlation between breastfeeding self-efficacy and breastfeeding duration and continuation [24].

Although GDM has a high prevalence in Saudi Arabia [25], the breastfeeding experience of mothers with GDM, including breastfeeding self-efficacy, is not commonly studied in Saudi Arabia. This study aimed to measure breastfeeding self-efficacy and identify self-efficacy determinants among Saudi women with GDM.

## 2. Materials and Methods

### 2.1. Study Design and Setting

We conducted a quantitative, observational, cross-sectional study, targeting pregnant women with GDM undergoing follow-up consults in GDM antenatal care clinics in King Saud University Medical City (KSUMC), Riyadh, Saudi Arabia. The study was conducted between January 2021 and April 2021.

### 2.2. Sample Characteristics

The equation *n* = $\frac{Z_{\alpha }{}^{2}P\left(1-P\right)\;}{d^{2}}$ was used to calculate the sample size, with *n* = sample size, Z_α_ = 1.96 indicating 95% confidence level, d = 0.05 indicating 5% precision, and *p* = 0.243 indicating 24.3% prevalence of GDM in Saudi Arabia [4]. The calculated sample size was 181 patients.

A pilot study was conducted on 20 expectant mothers with GDM before starting the study. This aimed to assess survey clarity and was not included in the analysis. The study inclusion criteria were Saudi pregnant women with gestational diabetes mellitus (GDM). Exclusion criteria were pre-existing diabetes mellitus, multiple pregnancies, and infants with congenital abnormalities.

### 2.3. Variables and Assessment Tool

We developed a questionnaire containing three parts: (1) sociodemographic data, (2) GDM-related knowledge, and (3) prenatal breastfeeding self-efficacy scale (PBSES).

The independent variables included the sociodemographic data, which showed correlations with the breastfeeding self-efficacy scale in the literature [1,13,26], such as age, BMI, level of education, mother’s occupation, husband’s monthly income, place of residence, smoking status, and chronic diseases other than GDM parity status. Other factors that showed correlations in the literature were included [9,13,21,22,27] such as previous breastfeeding experience, satisfaction with previous breastfeeding experience, previous breastfeeding duration, inquiry about the intention to breastfeed the coming newborn, and health education sources. In the second part, which contained GDM-related knowledge, questions were included from a valid tool used previously in a Saudi study [28]. It contains six items with binary responses. A score of 1 was given for the correct answer, whereas an incorrect answer was given a score of 0. A total score of 3 or more was categorized as high knowledge, while a score below 3 was categorized as low knowledge.

The prenatal breastfeeding self-efficacy scale (PBSES) was developed and validated: it contains 20 items to assess a mother’s perceived ability to breastfeed [29]. It was chosen as an instrument because of its approved validity [23] and valid Arabic version [24]. The 20 items were categorized into 4 groups based on the factors assessed. The first 7 items were designed to determine the abilities and skills of a mother to breastfeed in correlation with other life demands. The second group contained five items related to the knowledge of how to breastfeed. The third group, consisting of four items, focused on the uncomfortable feeling when the mother breastfeeds around people. The fourth group, with two items, was related to social pressure on breastfeeding mothers: this last group was independent and related to convincing their partners about the importance of breastfeeding and their ability to breastfeed for one year [29]. The overall scores ranged from 20 to 100. Higher scores indicate greater breastfeeding self-efficacy.

The Arabic version of the PBSES was used in this study. It was validated in a study conducted in Saudi Arabia [19] to assess breastfeeding self-efficacy in pregnant women. Cronbach’s alpha coefficient for the Arabic-translated prenatal breastfeeding self-efficacy scale was 0.83, suggesting that the items have high internal consistency [24]. Consent to use the Arabic version was obtained from the study’s author.

### 2.4. Ethical Approval

Ethical approval was obtained from the Institutional Review Board (IRB) committee of King Saud University College of Medicine (Project No. E-20-5422). Informed consent was explicit and indicated the purpose of the study and the participant’s right to withdraw without any obligation to the study team.

### 2.5. Statistical Analysis

Data were analyzed using IBM SPSS Statistics for Windows, version 26.0 (IBM Corp., Armonk, NY, USA). Descriptive statistics (mean, standard deviation, frequencies, and percentages) described the quantitative and categorical variables. Cronbach’s alpha coefficient was used to analyze the validity and reliability of the data. Cronbach’s alpha value was 0.912. A normality test was conducted. Differences in breastfeeding self-efficacy among sociodemographic characteristics and other variables were analyzed using a one-sided independent sample t-test or one-way ANOVA. Significant variables (*p* < 0.05) were included in a multiple linear regression analysis to investigate the predictors of breastfeeding self-efficacy. The absolute value of the Pearson correlation showed no evidence of multicollinearity. The fitted regression model was R= 0.626; R^2^ = 0.392; model fit: F = 8.136 *p* ≤ 0.001; statistically significant (enter method).

## 3. Results

A total of 145 expectant mothers with GDM completed the questionnaire, with a response rate of 80.1%. Table 1 shows the sociodemographic characteristics of the participants. The mean age was 32 years (range: 22–46 years). All participants were married, and 46.9% were multiparous. Only about a quarter of participants (24.3%) had normal BMI, 67.6% had a bachelor’s degree, and 38.6% were employed. Most of them (95.9%) had the intention to breastfeed. The majority had previous breastfeeding experience (70.3%), and about half (49%) had a previous breastfeeding duration of less than six months.

Table 1 shows the correlation between the overall prenatal breastfeeding self-efficacy score and the other independent variables. Participants with a post-graduate degree (*p* < 0.003), expectant mothers who had a previous breastfeeding experience (*p* = 0.005), those who previously had satisfactory breastfeeding experience (*p* < 0.001), mothers with a breastfeeding duration of 6 months or more (*p* < 0.001), and mothers who had the intention to breastfeed (*p* = 0.001) were more likely to have a higher PBSES score. In addition, expectant mothers who received education about GDM and breastfeeding (*p* = 0.005) and high knowledge scores (*p* = 0.002) were significantly correlated with the PBSES score. The correlation was positive.

The PBSES items, their means, and standard deviations are listed in Table 2. The item “I can breastfeed my baby around people I do not know” had the lowest mean item score of 2.01 ± 1.364. Meanwhile, the item “I can breastfeed my baby when my partner is with me” had the highest mean score of 4.21 ± 1.160. The total mean score was 64.07 ± 16.30 (range: 20–100).

**Figure 1:**
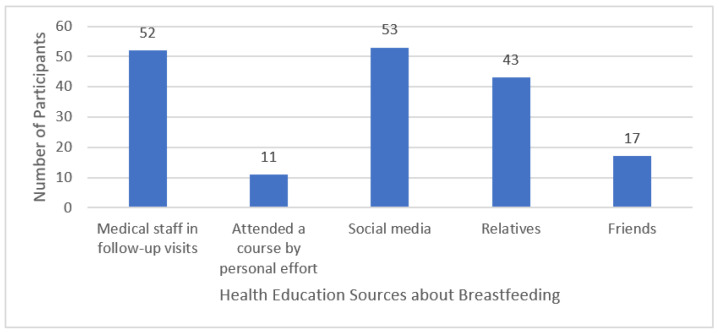
Health education sources about breastfeeding of the participants.

Table 3 shows the participants’ knowledge of GDM. The mean knowledge score was 2.28 ± 1.72 (range 0–6). Figure 1 demonstrates the health education sources about breastfeeding information: the main sources for breastfeeding information were social media (36.6%) and medical staff during follow-up visits (35.9%). However, some participants reported getting information from their relatives (29.7%) and friends (11.7%). Around a quarter of the participants (26.9%) claimed that they did not receive any information.

Table 4 shows that the regression analysis revealed three variables that explained 62.6% of the variance in breastfeeding self-efficacy scores among expectant mothers. Mothers who previously had satisfactory breastfeeding experience (β=0.218, *p* = 0.020), mothers who had a breastfeeding duration of 6 months or more (β=0.197, *p* = 0.036), and mothers with higher knowledge scores (β=0.259, *p* = 0.004) were more likely to have a higher PBSES score.

## 4. Discussion

Saudi Vision 2030 urges the Saudi Ministry of Health (MOH) to implement several health initiatives, including maternal and child health initiatives. The Ministry of Health has an advocative role in safe child feeding, including promoting breastfeeding.

Given the high prevalence of GDM in Saudi Arabia [4], which has frequently been reported as a factor that negatively affects breastfeeding experience [12], in-depth exploration of this issue can help plan effective breastfeeding promotion and counseling services.

A study conducted in 2016 that measured the association between GDM and exclusive breastfeeding at hospital discharge showed that the exclusive breastfeeding rate was 62.2% among mothers with GDM versus 75.4% among mothers without GDM [30].

The current study showed a mean total score of the prenatal breastfeeding self-efficacy scale of 64.07 ± 16.30, which is lower than other Saudi expectant mothers without GDM, who reported a mean score of 70 [24].

This study found that a higher academic level significantly influenced the prenatal breastfeeding self-efficacy scale score. Previous studies have supported a positive correlation between mothers with higher education levels and successful exclusive breastfeeding [31]. However, a previous study focusing on the correlations of mothers’ breastfeeding self-efficacy during the first three months of delivery showed no significant difference between maternal education and breastfeeding self-efficacy scores [32]. Other sociodemographic characteristics showed no significant relationship with self-efficacy scores.

This study showed that satisfactory previous breastfeeding experience predicts a higher breastfeeding self-efficacy score, as reported in the literature. A previous study showed that breastfeeding self-efficacy is higher among mothers with previous successful breastfeeding experience; however, mothers with previous challenging breastfeeding experiences had lower self-efficacy, which supports that the nature of the previous breastfeeding experience has a high impact on self-efficacy [33]. According to the infant feeding survey, mothers with previous successful breastfeeding experience for six weeks or more had a higher breastfeeding initiation rate [34]. Mothers who had prior experience of long-duration breastfeeding are more aware of the importance and benefits of breastfeeding physically and psychologically for their infants and themselves [35] and are thus more confident in succeeding instances.

This study found that a high knowledge score on breastfeeding and GDM predicts a higher breastfeeding self-efficacy score. The literature shows that mothers with GDM have less breastfeeding knowledge than other expectant mothers. Women with GDM were less likely to say that breastfeeding was the best way to feed infants [11]. A study conducted in Bangladesh showed that participants who lacked knowledge of GDM were less likely to have the intent to breastfeed [36].

A Korean study showed that breastfeeding intention after childbirth was correlated with a stronger perceived benefit of breastfeeding and higher levels of self-efficacy [17]. Notably, a study conducted in Saudi Arabia reported a high prevalence of poor awareness and knowledge about GDM [28]. Thus, antenatal care for breastfeeding in mothers with GDM should provide more accurate information on GDM and breastfeeding.

The high number of expectant mothers who rely on social media to obtain information about breastfeeding, with only a third of expectant mothers obtaining information through healthcare workers in the follow-up visits, raises questions about the implementation of breastfeeding counseling in routine antenatal visits. Expectant mothers who attended antenatal breastfeeding classes had significantly increased breastfeeding at six months [37].

Around a quarter of the participants claimed that they did not receive any information about breastfeeding. Education and training of all healthcare providers about breastfeeding support are essential components of breastfeeding support programs, including the Baby-Friendly Hospital Initiative [38]. A study that presented a breastfeeding care model showed that nurses have positive breastfeeding attitudes and self-confidence in providing breastfeeding education following training [39]. It was reported that different education methods were effective in promoting breastfeeding in antenatal and postpartum care [37]. Mothers in one study valued breastfeeding information and highlighted the need for education of family members and society [40].

A randomized controlled trial was conducted to promote exclusive breastfeeding, including an arm receiving two prenatal group sessions, an information package with breastfeeding images, and text messages until eight weeks postpartum, compared with a trial with standard care. Significantly higher breastfeeding self-efficacy scores were found in the intervention group (mean 62.46) than in the control group (mean 50.74) [41].

This study had several limitations. The study was conducted during the COVID-19 pandemic lockdown and restrictions, with a significantly decreased number of hospital visitors. Additionally, most of the recruited patients from the GDM clinics were excluded because they had one or more of the exclusion criteria, such as pre-existing diabetes mellitus, multiple pregnancies, or infants with congenital abnormalities. Exploring potentially confounding factors that can affect the intention to breastfeed requires a qualitative assessment and possibly a cohort follow-up to determine mothers’ actual breastfeeding practices in the postpartum period.

## 5. Conclusions

The breastfeeding self-efficacy score was lower among expectant mothers with GDM than in other pregnant populations, especially those with unsatisfactory previous breastfeeding experience and low knowledge scores about breastfeeding and GDM. In-depth and systematic health education should be conducted to improve the breastfeeding rate of pregnant women with GDM. Establishing systematic education about breastfeeding during antenatal care to improve breastfeeding experience and improve GDM outcomes is recommended. A qualitative study can explore this issue in-depth and provide more important data to help plan and implement care for such a group.

## Figures and Tables

**Table 1:** The socio-demographic characteristics of the participants and its correlation with the total prenatal breastfeeding self-efficacy score (*n* = 145).

Characteristics	Frequency (*n*)	Percentage (%)	Mean	SD	*p*-Value
Age (Range 22–46)					0.987
Less than 30	52	35.9	64.10	17.58	
30–35	48	33.1	63.79	15.69	
More than 35	45	31.0	64.33	15.77	
BMI					0.148
Underweight	0	0			
Normal	35	24.2	66.26	16.32	
Overweight	47	32.4	60.42	16.52	
Obese	63	43.4	61.91	15.07	
Level of education					0.003 *
High school or below	27	18.6	56.89	18.06	
Bachelor’s degree	98	67.6	64.18	15.04	
Post-graduated **	20	13.8	73.20	15.88	
Mother’s occupation					0.390
Student	10	6.9	70.20	20.42	
Employed	56	38.6	64.64	13.94	
Un-employed	79	54.5	62.89	17.29	
Husband’s occupation					0.965
Employed	140	96.6	64.12	16.42	
Un-employed	2	1.4	65.00	18.38	
Retired	3	2.1	61.67	14.22	
Monthly income					0.214
More than 20,000	19	13.1	66.11	14.78	
15,000–20,000	24	16.6	69.79	16.19	
10,000–14,000	48	33.1	64.17	11.65	
5000–9000	34	23.4	61.62	19.44	
Less than 5000	20	13.8	59.20	20.41	
Place of residence					0.475
City	141	97.2	64.20	16.40	
Village	4	2	58.25	15.52	
Smoking status					0.428
Non-smoker	144	99.3	63.97	16.32	
Past smoker	1	0.7	77.00	0	
Smoker	0	0	0	0	
Chronic disease					0.211
Yes	18	12.4	59.56	11.92	
No	127	87.6	64.70	16.77	
No. of previous pregnancies (Parity)					0.034 *
0	24	16.6	65.92	15.09	
1	28	19.3	58.79	14.41	
2	25	17.2	59.04	19.33	
3 or more **	68	46.9	67.44	15.55	
Previous breastfeeding experience					0.005 *
Yes **	102	70.3	66.50	14.94	
No	43	29.7	58.30	18.05	
Satisfaction with previous breastfeeding experience					<0.001 *
Primigravida	35	24.1	-	-	
Satisfied **	61	42.1	70.56	15.30	
Neither satisfied nor dissatisfied	40	27.6	59.73	11.76	
Dissatisfied	9	6.2	51.11	21.07	
Previous breastfeeding duration					<0.001 *
Did not breastfeed	35	24.1	58.14	18.98	
Less than six months	71	49.0	61.70	13.19	
Six months or more *	39	26.9	73.69	15.06	
Intention to breastfeed					0.001 *
Yes **	139	95.9	64.96	15.42	
No	6	4.1	43.33	23.50	
Knowledge level					0.002 *
High **	56	38.62	69.27	14.59	
Low	89	61.38	60.80	16.55	
Health education					0.005 *
Yes **	106	73.10	66.33	16.11	
No	39	26.90	57.92	15.39	
Total	145	100			

* Statistically significant (*p* < 0.05). ** Possible contributor factor.

**Table 2:** Descriptive statistic of prenatal breastfeeding self-efficacy score (PBSES) score by mean and standard deviation (*n* = 145).

PBSES	Mean	Standard Deviation
I can make time to breastfeed my baby even when I feel busy	2.90	1.257
I can breastfeed my baby even when I am tired	2.94	1.355
I can schedule my day around the breastfeeding of my baby	2.70	1.266
I can breastfeed my baby when I am upset	2.95	1.411
I can breastfeed my baby even if it causes mild discomfort	3.47	1.259
I can use a breast pump to obtain milk	2.97	1.467
I can prepare breast milk so others can breastfeed my baby	2.59	1.570
I can find out what I need to know about breastfeeding my baby	3.49	1.173
I can find the information I need about problems I have breastfeeding my baby	3.34	1.324
I know who to ask if I have any questions about breastfeeding my baby	3.33	1.286
I can call a lactation counselor if I have problems breastfeeding	2.84	1.398
I can talk to my healthcare provider about breastfeeding baby	2.79	1.343
I can breastfeed my baby when my family or friends are with me	3.08	1.436
I can breastfeed my baby around people I do not know	2.01	1.364
I can breastfeed my baby when my partner is with me	4.21	1.160
I can breastfeed my baby without feeling embarrassed	3.43	1.378
I can choose to breastfeed my baby even if my partner does not want me to	3.84	1.245
I can choose to breastfeed my baby even if my family does not want me to	4.08	1.228
I can talk to my partner about the importance of breastfeeding baby	4.17	1.124
I can breastfeed my baby for one year	2.93	1.480
Overall score	64.07	16.30

**Table 3:** Knowledge about gestational diabetes mellitus (GDM) of the participants and its correlation with the total prenatal breastfeeding self-efficacy Score (*n* = 145).

Knowledge	Frequency (*n*)	Percentage (%)
GDM always disappear after delivery without any consequences		
Yes	63	43.4
No	82	56.6
GDM may make the newborn develop obesity, diabetes, and cardiovascular diseases more likely than his/her peers		
Yes	46	31.7
No	99	68.3
Breastfeeding reduces the susceptibility of a newborn to a mother with GDM to develop obesity, diabetes, and cardiovascular disease		
Yes	51	35.2
No	94	64.8
Women with GDM more likely to develop type 2 diabetes, obesity, and cardiovascular disease		
Yes	74	51.0
No	71	49.0
Breastfeeding reduces the susceptibility of a mother with GDM to obesity, diabetes, and cardiovascular disease		
Yes	43	29.7
No	102	70.3
Breastfeeding within the first hour of birth reduces the risk of the newborn to a mother with GDM for hypoglycemia		
Yes	34	23.4
No	111	76.6
Mean knowledge score	2.28 ± 1.72	

**Table 4:** Predictors of breastfeeding self-efficacy among expectant mothers with gestational diabetes mellitus (GDM) (*n* = 145).

Predictor	Regression Coefficients	t	*p*-Value
Literacy level	0.141	1.640	0.104
Parity	0.060	0.752	0.454
Previous Breastfeeding	0.131	1.455	0.149
Satisfaction with Previous Breastfeeding Experience	0.218	2.363	0.020 *
Longer Previous Breastfeeding Duration	0.197	2.126	0.036 *
Intention To Breastfeed	0.024	0.283	0.777
Health education	0.111	1.392	0.167
Knowledge Score	0.259	2.936	0.004 *

Summary of model: R = 0.626; R^2^ = 0.392; model fit: F = 8.136 *p* ≤ 0.001; statistically significant (enter method). * Significant predictor.

## Data Availability

The datasets generated and analyzed during the current study are available from the corresponding author on reasonable request.

## Abbreviations

PBSES	prenatal breastfeeding self-efficacy score
GDM	gestational diabetes mellitus
SA	Saudi Arabia
